# Family planning interventions across the League of Arab States: a regional scoping review

**DOI:** 10.1093/inthealth/ihaf055

**Published:** 2025-05-15

**Authors:** Basil H Aboul-Enein, Suha Ballout, Patricia J Kelly

**Affiliations:** College of Arts and Sciences, Health and Society Program, University of Massachusetts Dartmouth, 285 Old Westport Rd., North Dartmouth, MA 02747, USA; Faculty of Public Health and Policy, London School of Hygiene and Tropical Medicine, 15-17 Tavistock Place, London WC1H 9SH, UK; Manning College of Nursing and Health Sciences, University of Massachusetts Boston, 100 William T. Morrissey Blvd., Boston, MA 02125 USA; College of Nursing, Thomas Jefferson University, 901 Walnut St., Philadelphia, PA 19107, USA

**Keywords:** Arab women, family planning, Middle East, North Africa, reproductive health

## Abstract

Family planning is crucial in improving maternal and child health, reducing fertility rates and promoting gender equity. Despite global advancements in contraceptive access, disparities persist across the League of Arab States due to sociocultural, economic and policy barriers. Understanding the effectiveness of existing family planning interventions in this region is essential for addressing unmet needs and guiding policy improvements. This scoping review aims to identify and appraise family planning interventions conducted across the League of Arab States. A comprehensive search of 14 databases was conducted using the Preferred Reporting Items for Systematic Reviews and Meta-Analyses for Scoping Reviews guidelines, focusing on studies published between 2010 and December 2024. The review included intervention-based studies that assessed family planning outcomes in Arabic-speaking countries. Data were extracted and tabulated. Seventeen intervention studies were identified, with the majority conducted in Jordan and Egypt. Effective interventions included pharmacist-led education, multisectoral collaborations, behavioural economics approaches and crisis-responsive strategies. While most studies reported positive effects on contraceptive uptake and knowledge, challenges such as low male engagement, provider resistance and sustainability concerns persisted. Interventions in crisis-affected settings demonstrated adaptability, but scalability remains a key issue. Future efforts should focus on culturally tailored strategies, long-term intervention sustainability and integrating family planning with broader health and economic empowerment programs.

## Introduction

Family planning is recognized as one of the 20th century's most significant public health achievements, contributing to improved maternal and child health, economic development and gender equity.^1^ The historical trajectory of family planning in low- and middle-income countries is closely tied to governmental policies. By the mid-20th century, many nations in the Arab region, like others globally, faced declining mortality rates without a corresponding decrease in fertility rates, leading to rapid population growth.^2,3^ In response, the United Nations Fund for Population Activities (UNFPA) was established in 1969, facilitating the implementation of family planning programs worldwide. Despite decades of investment in reproductive health, family planning remains an unmet need for >200 million women globally.^4^

However, in the Arab region, these global trends have intersected with deeply rooted cultural and religious values, creating a unique landscape for family planning. In many communities, particularly in rural or conservative areas, traditional family planning methods such as lactational amenorrhea, withdrawal and fertility awareness–based methods have historically been more prevalent. These methods were often chosen based on cultural preferences and religious teachings, with limited access to or acceptance of modern contraceptive methods due to stigma, religious views or lack of awareness. The introduction of modern contraceptive methods has faced various challenges in this region, influenced by gender norms and societal expectations regarding fertility. For example, in many communities, large families are seen as a symbol of status, while women's roles are often perceived as being closely tied to motherhood. These sociocultural expectations shape the acceptance and uptake of family planning interventions, particularly among women and men in rural, less educated or economically disadvantaged settings. Furthermore, there is often resistance to family planning programs from male partners or other influential family members, such as mothers-in-law, whose approval is essential in family planning decisions. These factors, coupled with regional disparities such as varying educational levels, urban versus rural differences and political instability, make the implementation of family planning programs complex and multifaceted. Family planning education initiatives are also affected by these cultural norms, with varying levels of success across different areas of the Arab region. In urban centres, where access to healthcare services is better, modern contraceptive uptake is relatively higher, while in rural or remote areas, traditional methods still dominate. Additionally, family planning efforts in conflict-affected countries face unique barriers, such as displacement, lack of access to healthcare infrastructure and limited outreach due to security concerns. These sociocultural, economic and political factors must be considered in the design and implementation of family planning programs to address unmet needs effectively.

Historically, women worldwide, including in the Arab region, have relied on traditional contraceptive methods such as lactational amenorrhea, withdrawal (coitus interruptus) and fertility awareness–based methods to regulate fertility. These methods were often preferred in areas where access to modern contraceptives was limited, particularly in rural or conservative communities. In the Arab region, the use of these methods was deeply intertwined with cultural and religious beliefs surrounding fertility, family size and gender roles. As a result, modern contraceptive methods have faced various challenges in acceptance, particularly in regions where sociocultural and religious factors strongly influence reproductive health practices.

Countries in the League of Arab States represent a heterogeneous demographic and socio-economic landscape, shaping family planning access and contraceptive use in distinct ways. Oil-rich nations, such as Qatar, the United Arab Emirates and Kuwait, have relatively small indigenous populations supplemented by large migrant worker communities. In contrast, Egypt's population exceeds 117 million, constituting approximately 23% of the Arab League population.^5,6^ The countries’ unique sociocultural, religious and policy landscapes contribute to uneven contraceptive uptake. Some Arab countries, including Morocco, Libya and Lebanon, report contraceptive rates of <20%, in contrast to overall Egyptian rates of >90%.^5^ This variability in contraceptive prevalence rates underscores the need for a comprehensive assessment of what interventions work best, for whom and under what conditions.^5^ These regional disparities, rooted in sociocultural and religious contexts, were key considerations in selecting studies for this scoping review. Studies that assessed family planning interventions in diverse sociocultural settings within the Arab region were prioritized to reflect the varied challenges and successes in contraceptive access and use. Despite growing global efforts to expand contraceptive access, the effectiveness of family planning interventions in the Arab region remains underexplored. Therefore, this scoping review seeks to identify and appraise family planning interventions implemented across the League of Arab States, identify effective strategies and highlight persistent barriers to contraceptive access. The League of Arab States presents a highly diverse context shaped by varying religious beliefs, socio-economic conditions, political stability and gender norms. These factors distinctly influence contraceptive access and family planning interventions across the region. For example, high-income Gulf countries, conflict-affected nations and North African states face different health system capacities and sociocultural dynamics, necessitating regionally tailored strategies.

## Methods

### Literature search

**Figure 1.**
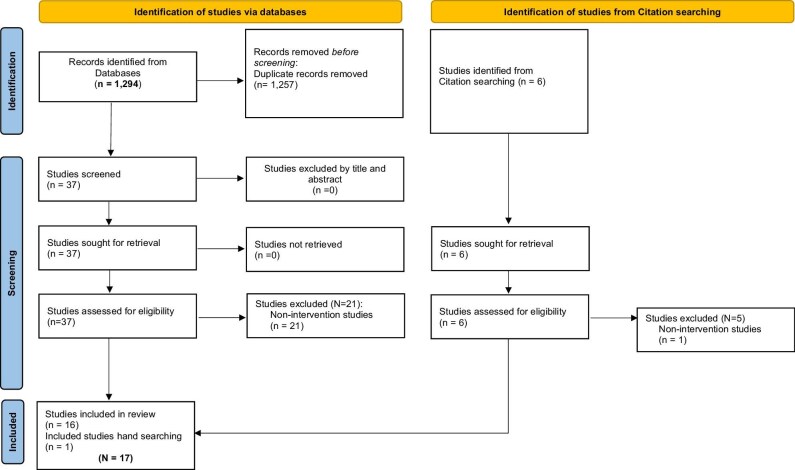
2020 PRISMA-ScR flow diagram.

A scoping review of the literature was conducted across 14 electronic databases using the Preferred Reporting Items for Systematic Reviews and Meta-Analyses for Scoping Reviews (PRISMA-ScR) and Arksey and O’Malley framework.^7,8^ The databases were selected based on their medical and biomedical scope for coverage of disciplines central to this review, namely, family planning, reproductive health, public health and global health, with a specific focus on capturing studies relevant to the Arab region. These included both medical databases (e.g. PubMed, Scopus) and regionally indexed sources to ensure contextual and geographical relevance. Search terms included keywords and Medical Subject Headings (MeSH) related to family planning, pregnancy, postpartum care and Arab women. Boolean operators (AND, OR) were applied to refine the search across different databases (see Table 1). The search covered all 22 Arabic-speaking countries in the League of Arab States as defined by Blair et al.,^9^ including nations from North Africa, the Levant and the Gulf region (see Table 1). The search strategy (see Figure 1) was adapted for each database, considering differences in indexing systems and controlled vocabularies (see Table 2). To ensure comprehensiveness, reference lists of all retrieved articles were manually screened for additional relevant studies. All articles retrieved were assessed against predefined eligibility criteria, ensuring relevance to the review's objectives.

**Table 1.** PICOS criteria for inclusion and exclusion of studies.

Parameter	Inclusion criteria	Exclusion criteria
Date range	Between 2010 and December 2024	N/A
Population	Adult populations residing in the League of Arab States (22 countries)^a^	Arab diaspora
Intervention type	Any form of non-pharmacological intervention that address family planning-related outcomes, including: community-based programs, family-based programs, mHealth/digital tools, educational interventions and environmental interventions	Pharmacological interventions alone Interventions that do not address family planning-related outcomes
Comparators	Control: received no intervention Received partial intervention (e.g. educational intervention only vs multicomponent intervention)	N/A
Outcomes of interest	Changes in family planning-related outcomes, i.e. knowledge, attitudes, beliefs etc. Changes in family planning referrals	Non-family planning–related outcomes
Language	English, Arabic or French	All other languages
Study type	Intervention studies with measured outcomes Peer-reviewed original research articles Original research conference publications	Non-numeric/categorical assessments or qualitative studies Non-peer-reviewed articles Study protocols Commentaries Narratives Communications Non-intervention studies White papers Similar article types Grey literature Thesis/dissertation

N/A: not applicable; PICOS: Population, Intervention, Comparison, Outcomes and Study.

a Algeria, Egypt, Bahrain, Comoros, Djibouti, Iraq, Jordan, Saudi Arabia, Kuwait, Lebanon, Libya, Mauritania, Morocco, Oman, Occupied Palestinian Territories, Qatar, Yemen, Somalia, Sudan, Syria, Tunisia and the United Arab Emirates.

**Table 2.** Electronic databases used with relevant search period and terms

Databases	Search period	Keywords, search terms and phrases
ArticleFirst, Biomed Central, BioOne, BIOSIS, CINAHL, EBSCOHost, ProQuest, PubMed, SAGE Reference Online, ScienceDirect, Scopus, SpringerLink, Taylor & Francis and Wiley Online	2010–December 2024	(All fields) ‘reproductive health’ OR ‘family planning’ OR ‘contraception’ AND (All fields) ‘intervention’ OR ‘program’ or ‘programme’ OR ‘promotion’ OR ‘promoting’ AND (All fields) ‘Algeria’, ‘Egypt’, ‘Bahrain’, ‘Comoros’, ‘Djibouti’, ‘Iraq’, ‘Jordan’, ‘Saudi Arabia’, ‘Kuwait’, Lebanon’, ‘Libya’, ‘Mauritania’, ‘Morocco’, ‘Oman’, ‘Palestinian Territories’, ‘Qatar’, ‘Yemen’, ‘Somalia’, ‘Sudan’, ‘Syria’, ‘Tunisia’, ‘United Arab Emirates’

### Eligibility criteria

This review focused on peer-reviewed, intervention-based studies published between 2010 and December 2024 that assessed family planning–related outcomes in Arabic-speaking countries. Only studies that evaluated measured outcomes were included to ensure methodological rigor and relevance. Eligible studies examined interventions targeting family planning as a primary focus or as part of a multibehavioural intervention at the individual, community, family or policy level. The review specifically included studies conducted with pregnant women and/or mothers who received education, training or other structured interventions related to family planning. Only English, Arabic or French articles were considered for inclusion, reflecting the dominant languages used in academic and public health publications across the League of Arab States. This multilingual approach aimed to reduce language bias and enhance the representativeness of regional studies. While grey literature and unpublished reports were excluded to maintain methodological rigor, we acknowledge this decision may have limited the comprehensiveness of the review. Future research could benefit from incorporating grey literature and expert consultations to mitigate publication bias and capture non-peer-reviewed programmatic data. Studies focused on populations outside the League of Arab States or the Arab diaspora were excluded. Protocol studies, brief communications and grey literature—including non-peer-reviewed reports, conference abstracts and government documents—were not considered. This decision ensured that all included studies met peer-reviewed standards and provided empirical evidence on family planning interventions. Applying these eligibility criteria, this review aimed to capture rigorous, context-specific research that informs family planning interventions in the Arab region (see Table 2).

### Study selection and data extraction

One author independently conducted the literature search and selected studies for inclusion. Differences were discussed with the research team to reach a consensus; a second author resolved discrepancies if needed. Rayyan (Cambridge, MA, USA) and Qatar Computing Research Institute (Dohan, Qatar) software was used to assist in the screening process and study selection titles.^10^ Three of the authors extracted and tabulated data. When the list of selected studies was finalized, one author extracted and cross-checked the following for each survey: author, date, target population, country, type of study, sample size, type, intervention details, measured parameters, main results and main recommendations (see Table 3). Differences of opinion in data extracted were discussed to reach a consensus and tabulated. Since methodological quality assessment is not a prerequisite for scoping reviews, we did not appraise the included studies.^11^

**Table 3.** Summary of findings and study characteristics (N=17)

Author (year), country	Goal	Target population/sample size	Type of study	Intervention details	Results	Recommendations
Akour et al. (2017)^12^, Jordan	Examine effect of pharmacist-provided information booklet on increasing knowledge of Jordanian women about safe/effective OCP use	160 women who had used OCPs at least once; IG=80; CG: 80	RCT; KA assessed pre-, post-, 3 months post	IG: pharmacist-provided 6-page booklet on types of OCPs, mechanisms of action, precautions, instructions, potential drug–drug interactions, alternative contraceptive methods, explained to women by pharmacist in 10-minute session; CG: conventional counselling about OCP dosing	IG knowledge and attitude scores significantly increased (p=0.033; p=0.014); no change in CG	Results provide insight into vital role of pharmacist in Jordanian women's health care, specifically in ensuring safe/effective use of OCPs, assisting FP decision-making
Cooper et al. (2016)^21^, Egypt	Document results of integrated reproductive and maternal/child health community-based program in two regions of Egypt (Upper and Lower)	2454 households from 120 clusters in 4 districts; IG: 3049; CG:3058	Cluster RCT	Mobile clinic to provide primary care, training private pharmacists, increasing contraceptive availability at government health facilities	Contraceptive use: Upper Egypt, IG decrease 6 pp, CG decrease 15 pp in CG; Lower Egypt, IG, CG decrease 3 pp; Knowledge of optimal birth spacing: Upper Egypt, IG, increase 14 pp, CG increase 4 pp; Lower Egypt, IG, increase 26 pp, CG 27 increase pp; Reproductive intention: Upper Egypt, IG decrease 1 pp, CG decrease 3 pp; Lower Egypt, IG increase 11 pp, CG increase 4 pp; Contraceptive use decision-making: Upper Egypt, IG/CG increase 16/6 pp; Lower Egypt, IG and CG increase 15 pp	Intervention had a positive effect on knowledge of optimal birth spacing in one region, on wanting to delay next pregnancy in another and on pregnancy risk and joint decision-making in both
Curry et al. (2015)^27^, Djibouti, among other countries	Describe lessons learned during first 2.5 y of implementing the ongoing SAFPAC initiative	Catchment population of 698 053 reproductive age women	Program evaluation	Program supports government health systems to deliver FP services in crisis-affected settings; strategy focuses on four broad interventions drawn from public health best practices in stable settings: competency-based training for providers, improved supply chain management, regular supervision and community mobilization to influence FP norms/attitudes; specifics include use of data to inform decision-making, training new providers and monthly supervision with simple checklists	Initiative reached 52 616 new users of modern contraceptive methods across the five countries, 61% of whom chose long-acting methods of implants or intrauterine devices	Despite constraints in crisis-affected countries, it is feasible to extend access to a range of contraceptive methods, including long-acting reversible contraceptives, using best practice approaches established in more stable environments
Curry et al. (2015)^26^, Djibouti, among other countries		267 private OB/Gyn and general practitioners providing FP services; IG: 135; CG: 132				
Elden et al. (2019)^22^, Egypt	Assess impact of multisectoral intervention model on FP utilization at the PHC level	1247 married women from 10 PHC units (health sector–related intervention), 13 comparison PHCs	Quasi-experimental design	IG: health sector FP-related interventions (capacity building, of reallocation of human resources); CG: non-health sector interventions (religious training on FP, messages to religious institutions, use of teachers to disseminate messages, health education in schools, literacy classes)	At the district level, significant increase in new FP clients (p=0.006), a 43% change; mean number of IUDs dispensed significantly increased by 391% (p=0.002)	Mobilizing and optimizing resources use, empowering district authorities, strengthening collaboration across sectors were drivers in scaling up FP use
El-Khoury et al. (2015)^14^, Jordan	Evaluate effects of EBM program on providers’ KAP	267 providers	RCT	Provider seminar on DMPA and two 15-min one-on-one educational visits to reinforce seminar messages	Only 38% of groups 1 and 2 received both educational visits and attended a seminar; results found no program impact on knowledge; some evidence of improved attitudes and confidence; no change in DMPA-related practices	Low attendance from low demand, provider fatigue; negative DMPA attitudes compared with other FP methods; no evidence that EBM was effective in targeting unpopular FP method
El-Khoury et al. (2016)^13^, Jordan	Evaluate effects of involving men in family planning counselling	Women from 23 PHCs: group 1, women only counseling (n=417); group 2, couples counselling (n=416); group 3, no counselling (n=414)	RCT	Trained FP communication counsellors provided free FP vouchers to all participants; initial couples counselling visits scheduled to accommodate husbands’ work schedule	Compared with no counselling, couples counselling led to a 54% increase in uptake of modern methods, not significantly different from 46% increase from women-only counselling; in couples counselling arm, main reasons for not receiving counselling were husbands’ lack of availability (14.7%) and refusal to be counselled (6.7%)	Outcome due to low rates of compliance in couples’ group; more tailored approaches needed to increase men's participation; counselling in homes appeared most effective in moving users away from traditional toward MFP; changing FP non-users’ behaviours may require more tailored interventions
Ghosh and Thornton (2024)^15^, Jordan	Assess how the presence of the woman's mother-in-law impacts the effectiveness of an FP program	N=662 women, 457 husbands; three groups: women-only counselling (35%); couples counselling (32%); no counselling (33%)	RCT	Home-based FP counselling to women in the treatment groups either with or without husbands; home visits every 4–6 weeks, discussing the benefits of FP, birth spacing, modern methods, concerns, referrals to local FP providers, vouchers for free FP services	For women not living with their mother-in-law, both woman-only and couples counselling increased FP take-up by 7.4 and 16.6 pp; effect of couples counselling >2 times woman-only counselling (statistically significant); for women residing with mother-in-law, woman-only counselling significantly increased FP use 3 pp (>11% in control)	Women not living with mother-in-law respond both to woman-only and couples FP counselling; non-spousal family members can have important roles in determining the effectiveness of FP interventions
Hagag et al. (2022)^23^, Egypt	Measure FP dynamics (discontinuation/switching failure) rates among postpartum new FP users; assess role of follow-up counselling to decrease discontinuation	N=264 from two health centres	RCT	IG: follow-up counselling sessions every 3 months with messages on perfect use of each FP method, managing common side effects, advantages of contraceptive use; CG: observational follow-up	In IG after counselling, dramatic shift towards IUD Implanon (26.5–3.8%); significant difference between those who switched and did not switch by type of contraception (p=0.001); 37.5% of those discontinuing became users again by the end of counselling sessions; no change in CG; no significant difference between groups in FP dynamics	Intervention success was to improve FP reuse after discontinuation
Hutchinson and Meekers (2012)^24^, Egypt	Estimate effects of health communication campaign on precursors to FP use (e.g. spousal communication, birth spacing attitudes) and modern FP use	1708 females from seven villages exposed to community-based multimedia health communication campaign; 378 from two villages not exposed	Post-intervention panel survey data analysis	National integrated health communication campaign using television, radio, public affairs and press advertising; specific FP messages on birth spacing, need for postpartum FP	Fixed effects, instrumental variables estimator, controlling for unobserved heterogeneity, found large, statistically significant effect on modern FP use (p=0.043)	Difficulties evaluating FP communication programs may be surmountable using panel data and analytic methods to address both observed and unobserved heterogeneity in exposure
Kamhawi et al. (2013)^16^, Jordan	Enhance quality of FP services and increase percentage of women using MFPM	352 women post-FP clinic visit	Post-test	Multiple activities, e.g. technical, financial assistance to health centres, community outreach, home visits, plays, women's groups, training on family health for religious leaders, improved client–provider counselling based on the Consult and Choose approach with provider cue cards^a^	New clients increased to >23 000; 4/5 of women reported a high level of satisfaction with services that correlated to use of Consult and Choose materials; 81% used a MFPM	Limitation that evaluation did not follow up to assess whether use of Consult and Choose materials associated with decreased levels of discontinuation and unmet need, ultimate program goal
Komasawa et al. (2019)^17^, Jordan	Enhance capacity of village health centres to improve quality and quantity of FP services in rural Jordan	2061 married women from five village health centres	Endline surveys	Facility-based component included FP training for nurses or midwives, providing basic medical equipment, FP education and supplies, visits by maternal and child health supervisors and updating FP service manual; community-based component supported health committee in each village with workshops, action plan and monitoring; provided seed money for activities	IG had 6/8 primary indicators (e.g. use of FP services, participation in health activities, source of reproductive health information) with significant positive effects; no significant difference in secondary outcomes of MFPM between the two groups	Project had impacts on increased use of MFPM, husbands’ perception of FP; integration of facility/community-based approaches may be effective in shifting from traditional to MFPM in other rural areas
Prince et al. (2023)^18^, Jordan	Compare impact of two behavioural economics interventions on use of MFPMs	1032 women: IG1: 326, IG2: 411, CG: 295	Cluster RCT over 9 months	IG1 received behavioural economics–augmented FP counselling; IG2 received FP intervention, i.e. text messaging and augmented counselling based on framing/identity-priming behavioural economics principles	26.8% of women in CG, 42.1% of IG1 sand 45.2% of women in IG2 used MFPMs continuously for 9 months; pregnancy rate significantly higher in CG (13.7%) compared with IG1 (7.0%) and IG2 (7.4%)	Simple behavioural economics–based interventions can be effective methods for enhancing the use of MFPMs
Sagiron et al. (2024)^28^, Sudan	Assess impact of FP education program on utilization rate	456 reproductive-age women	Cluster RCT	20-h community-based education program about FP methods	Use of FP methods increased from 114 (50%) to 190 (83.3%) (p= 0.001); use of natural methods, oral contraceptives and injectables significantly increased from 5.2%, 26.2% and 5.3% to 15.4%, 34.2% and 15.8%, respectively	FP education program significantly increases the level of FP use
Tahaineh et al. (2020)^19^, Jordan	Assess effect of pharmacist-delivered educational intervention on women's oral contraceptive knowledge	179 women	Quasi-experimental pre/post design	45-min educational session on oral contraceptives delivered by a clinical pharmacist	At baseline, one-quarter of participants relied on other people's experiences as their main source of information on oral contraceptives; 28.1% considered oral contraceptives harmful; correct answers post-intervention increased significantly compared with pre-intervention (p<0.005)	Educational intervention introduced by clinical pharmacists improved the knowledge of women in Jordan regarding oral contraceptives
Tawab et al. (2021)^25^, Egypt	Evaluate two intervention models integrating FP into worker health and livelihood programs	Model 1: IG 1519 young men and women job-seeker participants, CG 1082 non-participants; model 2: IG 1958 workers in factories with peer educators, CG 1047 in factories with no educators	Pre/post evaluation	Model 1, FP/Livelihood Program: 5-d integrated FP/RH and livelihood training workshop (2.5 d for FP/RH, 2.5 d for livelihood skills), including basic FP/RH information and employment-finding skills; Model 2, FP/RH Program: trained peers delivered FP/RH messages to factory workers with factory nurse available for referrals to pharmacist and MD	Model 1: IG had improvements in seven of eight knowledge and attitude indicators, no significant change in current FP use; Model 2: no change in KAP, no change in current FP use	More research needed to demonstrate economic benefits of integrating FP/RH into livelihood and worker health programs, e.g. effect of relationships, gender attitudes and FP/RH partner communication
Underwood et al. (2013)^20^, Jordan	Assess effects of training program to enhance role of MRLs in promoting family welfare, general RH, FP	136 religious leaders	Pre/post panel study design	2-d, eight-session training workshops centred around the Manual on Family Health, which describes the role of MRLs in promoting family welfare, male and female relations in Islam, Islam and family health, safe motherhood and birth spacing, leadership skills, community mobilization for better health, FP methods, authentic Islamic sources for Prophet Mohammad's sayings/actions related to these topics^b^	Post-intervention, MRL cited more methods deemed acceptable with Islamic teachings (p<0.001), had more positive FP attitudes (p<0.05), preached/counselled on FP topics more frequently (p<0.01)	Culturally appropriate training for religious leaders can lead to a deeper understanding of and appreciation for RH and FP, with effects manifesting as increased preaching and/or counselling about these topics

FP: family planning; pp: percentage points; PHC: primary health care; IUD: intrauterine device; EBM: evidence-based medicine; KAP: knowledge, attitudes and practices; IG: intervention group; CG: comparison/control group; RH: reproductive health; MRL: Muslim religious leader.

^a^No specific theory, but informed by understanding that behaviour change is a function of beliefs, attitudes and intentions.

^b^Ideation—when referring to the constellation of cognitive, emotional and social factors associated with behavioural change, specifically in the area of family planning. Their key idea is that a shared language and geographic proximity allow ‘changing perceptions, ideas, and aspirations’ to be communicated with members of any given community.

While we focused on peer-reviewed studies to ensure methodological rigor, we acknowledge that excluding grey literature, including reports from organizations like the United Nations, may limit the comprehensiveness of this review. These organizations often hold valuable data that may not be published in peer-reviewed journals. Future scoping reviews could benefit from including grey literature to provide a more holistic view of family planning interventions in the Arab region.

## Results

A total of 17 intervention-based studies were identified across multiple Arab countries and crisis settings.

In Jordan, where nine studies were conducted, researchers examined a range of intervention strategies to enhance contraceptive knowledge, uptake and access.^12–20^ These interventions primarily focused on pharmacist-led education, male involvement in family planning counselling, community-based interventions and behavioural economics approaches to influence modern contraceptive use.

In Egypt, five studies assessed various family planning strategies, including community-based mobile clinics, multisectoral interventions at the primary healthcare level and national health communication campaigns.^21–25^ These studies highlighted positive effects on contraceptive decision-making, birth spacing knowledge and increased access to modern contraceptives, although challenges remained in sustaining usage and overcoming sociocultural barriers.

Crisis-affected settings including Djibouti^26,27^ were examined in two interventions, implemented under the CARE-funded Supporting Access to Family Planning and Post-Abortion Care in Emergencies (SAFPAC) initiative, focused on training providers, improving supply chains and mobilizing communities to increase access to long-acting contraceptive methods. Despite resource constraints, findings demonstrated that best practice approaches from stable environments could be successfully adapted to crisis settings.

Finally, one study conducted in Sudan assessed the impact of a community-based family planning education program.^28^ The intervention resulted in a significant increase in contraceptive uptake, particularly in the use of oral contraceptives and injectables, reinforcing the effectiveness of education-driven approaches in low-resource settings. These findings emphasize the diverse range of family planning interventions implemented across the Arab region, highlighting effective strategies, regional disparities and remaining gaps in contraceptive access and uptake.

The settings inside of the countries varied, with the largest number of interventions (n=6) conducted inside of the health sector, i.e. in pharmacies or clinical practices.^12,14,16,17,19,23^ Five studies used cluster designs that were district- or statewide.^18,20–22,28^ Two interventions were conducted in urban areas,^13,15^ two with samples from largely rural areas^24,25^ and two working in crisis settings.^26,27^

### Samples

The studies included in this review targeted a range of populations, including individual family planning users, healthcare providers, community groups and the broader public.

Seven studies focused on clients receiving services at family planning or primary care clinics, assessing interventions designed to improve contraceptive knowledge, uptake and decision-making.^12,13,16,18,19,22,23^ Other studies implemented community-based approaches, with one study delivering contraceptive interventions directly to households^15^ and another engaging women's groups within targeted communities.^28^ Additionally, one intervention integrated family planning education into workplace health programs for factory workers and job seekers,^25^ while another study assessed the impact of a community-wide health education campaign.^24^ At the service delivery level, four studies focused on strengthening contraceptive service capacity within primary care and family planning clinics.^17,21,26,27^ One study targeted healthcare providers, assessing an evidence-based medicine program for provider education on contraceptive methods.^14^ Finally, engagement with religious leaders as key influencers in reproductive health decision-making was explored in one study, which provided training on reproductive health topics in general and family planning specifically.^20^

### Intervention content

The interventions included in this review varied in scope, from method-specific educational programs to multicomponent community and health system interventions.

#### Method-specific educational intervention

Three studies focused on educational interventions targeting a specific contraceptive method. Akour et al.^12^ and Tahaineh et al.^19^ evaluated the impact of pharmacist-led education through booklets or direct counselling on women's knowledge, attitudes and use of oral contraceptive pills (OCPs). Similarly, El-Khoury et al.^14^ assessed an educational program for healthcare providers on depot medroxyprogesterone acetate (DMPA) to enhance provider knowledge and counselling on this method.

#### Counselling-based interventions

Three interventions emphasized counselling on modern family planning methods (MFPMs), covering their advantages, effectiveness, contraindications and side-effect management.^18,23,28^ Two additional studies incorporated family planning counselling within a broader household or partner-based approach. Ghosh and Thornton^15^ and El-Khoury et al.^13^ investigated the effectiveness of counselling women and their partners together, addressing spousal influence on contraceptive uptake.

#### Health system strengthening interventions

Four studies focused on improving service availability and strengthening health system capacity. Cooper et al.^21^ assessed the impact of mobile clinics, pharmacist training and increased contraceptive availability on family planning outcomes. Curry et al.^26,27^ implemented a comprehensive approach combining provider training, supervision, supply chain management and community education to improve access in stable and crisis-affected settings. Komasawa et al.^17^ evaluated interventions that enhanced provider training, ensured adequate contraceptive supplies, updated service manuals and strengthened village health committees to improve access in rural settings.

#### Multisectoral and community-based interventions

Two studies adopted multisectoral approaches, integrating contraceptive services into the health and education sectors. Elden et al.^22^ and Kamhawi et al.^16^ combined clinic-based interventions with provider training, reproductive health education in schools, literacy classes and religious leader training to expand access.

#### Family planning integrated into broader health and social programs

Three studies integrated contraception within broader reproductive health and social well-being initiatives. Tawab et al.^25^ combined family planning education with employment training and worker health initiatives, addressing reproductive health and economic empowerment. Hutchinson and Meekers^24^ examined a national health communication campaign that promoted birth spacing, spousal communication and modern contraceptive use. Underwood et al.^20^ integrated family planning into safe motherhood and family welfare messaging, emphasizing its role in maternal and child health.

#### Theoretical and behavioural frameworks applied in interventions

Four studies applied structured theoretical frameworks to guide their interventions. El-Khoury et al.^14^ utilized evidence-based medicine principles in their provider training. Kamhawi et al.^16^ focused on behavioural change theories, emphasizing that beliefs, attitudes and intentions shape contraceptive behaviours. Prince et al.^18^ incorporated behavioural economics principles into their intervention, leveraging identity priming and framing techniques to promote sustained modern contraceptive use. Underwood et al.^20^ applied ideation theory, which considers the cognitive, emotional and social influences on behaviour change, in designing a training program for Muslim religious leaders to promote family planning.

### Outcomes

The results across the 17 studies reviewed were largely positive, demonstrating improvements in family planning knowledge, contraceptive uptake and provider engagement. However, some interventions showed limited effects on sustained contraceptive use or provider adherence. Several interventions significantly enhanced family planning knowledge and attitudes. Akour et al.^12^ and Tahaineh et al.^19^ found that pharmacist-led counselling and educational materials resulted in statistically significant improvements in women's knowledge and attitudes toward oral contraceptive pills. Similarly, Tawab et al.^25^ reported increased knowledge from an employment-based family planning and reproductive health training program, though no improvement in contraceptive uptake was observed. Underwood et al.^20^ found that training Muslim religious leaders led to enhanced family planning attitudes and increased discussions on reproductive health topics in religious settings.

Among the eight studies assessing contraceptive use, seven reported an increase in the uptake of MFPM.^13,15,18,21,23,24,28^ Notably, behavioural economics–based interventions^18^ and counselling-based interventions targeting both women and their partners^13,15^ significantly increased modern contraceptive uptake. However, Komasawa et al.^17^ found no significant change in MFPM use despite positive effects on six of eight precursor indicators, including increased awareness and positive attitudes toward contraceptive use.

Four studies documented increased numbers of family planning clients following interventions to improve service access and availability.^16,22,26,27^ These studies also reported significant increases in MFPM adoption, particularly in interventions involving health system strengthening, provider training and community outreach.

The only study reporting no significant change in provider-related contraceptive practices was El-Khoury et al.,^14^ which evaluated a provider training intervention focused only on DMPA. The study suggested provider fatigue and negative attitudes toward this method contributed to the lack of impact. Similarly, despite knowledge gains, Tawab et al.^25^ found no changes in contraceptive use in one of their intervention models targeting the general health and economic livelihood of factory workers.

## Discussion

The findings of this scoping review highlight the diversity of family planning interventions implemented across the League of Arab States and the varying levels of success in addressing contraceptive knowledge, uptake and service delivery. While many interventions demonstrated positive effects, significant barriers and research gaps remain, particularly in terms of long-term contraceptive adherence, male involvement and intervention sustainability. The reviewed studies emphasize the effectiveness of educational and counselling-based interventions in improving contraceptive knowledge and attitudes among women. Pharmacist-led education programs in Jordan significantly improved women's understanding of oral contraceptives,^12,19^ while community-based interventions in Egypt enhanced birth spacing knowledge and reproductive decision-making.^21,24^ Also, training religious leaders in Jordan resulted in greater acceptance of modern contraceptive methods and increased discussions on family planning within religious contexts.^20^ Interventions that integrated behavioural economics approaches were particularly successful in sustaining contraceptive use. Prince et al.^18^ demonstrated that framing and identity-priming techniques led to more women continuously using modern family planning methods over 9 months. Similarly, partner-inclusive counselling in Jordan showed a significant increase in contraceptive uptake, although male involvement remained limited due to sociocultural constraints.^13,15^

Despite the positive outcomes of many interventions, several challenges persist in achieving widespread contraceptive uptake and sustainability. One key issue is low male engagement in family planning decision-making. Two studies in Jordan found that husbands’ reluctance to participate in counselling sessions negatively impacted modern contraceptive uptake, even when joint counselling was made available.^13,15^ Similarly, interventions that sought to engage non-spousal family members, such as mothers-in-law, found mixed results, suggesting that family dynamics play a crucial but underexplored role in contraceptive decision-making.^15^ Provider-related challenges also contributed to intervention limitations. While provider education programs aimed to improve contraceptive counselling and service delivery, results were mixed. A training initiative that focused solely on DMPA failed to impact provider knowledge or practice, likely due to negative provider attitudes and low demand for DMPA.^14^ This finding suggests that provider resistance to specific contraceptive methods may hinder family planning uptake, highlighting the need for behavioural interventions targeting healthcare providers.

Intervention in crisis-affected settings demonstrated that family planning services can be successfully implemented even in unstable environments. The SAFPAC initiative in Djibouti and other conflict-affected countries significantly increased contraceptive uptake, particularly in long-acting reversible contraceptives.^26,27^ This suggests that leveraging best practices from stable settings such as provider training, supply chain management and community mobilization can help expand contraceptive access in fragile contexts. Similarly, community-based education programs in Sudan led to a significant increase in contraceptive use, reinforcing the effectiveness of education-driven approaches in low-resource settings.^28^ However, scalability and long-term sustainability remain key concerns, as many pilot programs lack follow-up evaluations to assess continued contraceptive adherence and health system integration.

It is important to acknowledge that the data included in this scoping review may be inherently biased due to differences between participants and communities that are included in research studies versus those that are not. Studies often rely on samples from specific communities that may be more likely to participate in research, potentially overrepresenting certain populations and underrepresenting others, particularly those in more marginalized or rural settings. As a result, the findings of this review may not fully reflect the experiences or needs of all communities within the League of Arab States. This selection bias can impact the generalizability of the results and may limit the ability to draw conclusions that are broadly applicable to the entire region. Further research, including studies that capture a more diverse range of communities and utilize alternative sampling methods, is needed to address these gaps and ensure that family planning interventions are inclusive of all populations.

### Implications for policy and future research

This scoping review highlights several critical unmet needs in the family planning arena of the League of Arab States. Policymakers can focus on scaling up successful interventions, such as behavioural economics–based strategies and multisectoral collaborations that integrate family planning with employment, education and religious engagement.^20,22,25^ Also, efforts to increase male engagement in family planning can consider more tailored approaches, such as home-based counselling, flexible scheduling and culturally adapted messaging.^13,15^ These initiatives can enhance community outreach and foster cultural acceptability, improving contraceptive uptake across diverse settings. Furthermore, efforts to increase male engagement in family planning must be prioritized. Tailored approaches, including home-based counselling, flexible scheduling and culturally adapted messaging, can enhance male participation and support joint decision-making. The review also underscores the importance of integrating family planning with broader social programs, including health and economic empowerment initiatives, which could foster more sustainable change in reproductive health outcomes.

Tailored policy approaches are needed to reflect the unique socio-economic and political contexts across subregions of the Arab League. In North African countries such as Egypt and Morocco, where public sector infrastructure is relatively established but access disparities persist, policies should focus on expanding community-based services and strengthening provider training. In contrast, Gulf states like Qatar and the United Arab Emirates have robust health systems but face challenges in ensuring equitable access for large migrant populations, necessitating inclusive service models and employer-based outreach. Meanwhile, crisis-affected countries such as Sudan, Syria and Yemen require adaptive policies that prioritize mobile service delivery, supply chain resilience and humanitarian integration to maintain continuity of care. Recognizing these subregional variations is essential for designing effective, context-specific family planning interventions.

Scalability remains a key challenge across many interventions identified in this review, especially those that showed initial success in pilot or community-based programs. Sustaining these interventions at scale requires several conditions: long-term funding mechanisms, strong political will and health system integration. For example, expanding interventions beyond donor-funded pilots demands national ownership and budget allocations. Community engagement strategies, such as involving local leaders, religious figures and peer networks, can also enhance trust and ensure cultural relevance. Finally, embedding family planning services into existing primary healthcare systems and workforce development plans is essential to maintain consistent delivery, supervision and supply chain infrastructure. Policymakers should prioritize cross-sector partnerships and institutional frameworks that support scale-up without compromising quality or equity.

Future research needs to address long-term contraceptive adherence, particularly by evaluating the sustainability of interventions beyond the study period. More studies are needed on how family and community dynamics influence contraceptive decision-making and the economic benefits of integrating family planning into broader livelihood programs.^25^ Understanding the role of male partners and community leaders in these decisions will be critical in developing effective interventions. Additionally, research exploring the economic benefits of integrating family planning into broader livelihood programs, including education and employment, should be prioritized. Finally, further exploration of provider attitudes and biases is necessary to improve contraceptive counselling and service delivery in both stable and crisis-affected settings.

Research should also examine the integration of family planning services into national health systems to ensure they are accessible, sustainable and adequately resourced. This scoping review comprehensively assessed family planning interventions in the League of Arab States, highlighting successful strategies, persistent barriers and key research gaps. While many interventions demonstrated positive impacts on contraceptive knowledge and uptake, challenges such as low male engagement, provider resistance and sustainability concerns remain. Evidence-based, culturally responsive policies are needed to improve family planning outcomes and further research on long-term intervention effectiveness and integration into national health systems.

## Data Availability

All data generated and analysed in this review are included in the published review article.
